# S1PR2 Inhibition Attenuates Allergic Asthma Possibly by Regulating Autophagy

**DOI:** 10.3389/fphar.2020.598007

**Published:** 2021-02-10

**Authors:** Hanye Liu, Liangchang Li, Zhengai Chen, Yilan Song, Weidong Liu, Ge Gao, Li Li, Jingzhi Jiang, Chang Xu, Guanghai Yan, Hong Cui

**Affiliations:** ^1^ Jilin Key Laboratory for Immune and Targeting Research on Common Allergic Diseases, Yanbian University, Yanji, China; ^2^ Department of Pharmacology, Yanbian University College of Medicine, Yanji, China; ^3^ Center of Medical Functional Experiment, Yanbian University College of Medicine, Yanji, China; ^4^ Department of Anatomy, Histology and Embryology, Yanbian University College of Medicine, Yanji, China

**Keywords:** sphingosine-1-phosphate, S1P2, asthma, autophagy, RAC1/JNK pathway 2

## Abstract

This study is to investigate the role of Sphingosine-1-phosphate (S1P) in the asthma progression, and the involvement of autophagy. Airway remodeling mice were subjected to the HE, PAS, and Masson staining. Protein expression levels in the tissues, samples and model cells were detected with ELISA, Western blot analysis, and immunohistochemical/immunofluorescent analysis. The S1P2 receptor antagonist JTE-013 decreased the inflammatory cell infiltration and goblet cell production in asthmatic mice tissues. The IL-1, IL-4, IL-5 and serum IgE contents were decreased in bronchoalveolar lavage fluid, while the Beclin1 expression in lung tissues was decreased. The LC3B1 to LC-3B2 conversion was decreased, with increased P62 accumulation and decreased p-P62 expression. In airway remodeling mice, JTE-013 significantly decreased collagen deposition in lung tissues and decreased smooth muscle cell smooth muscle activating protein expression. In lung tissue, the expression levels of Beclin1 were decreased, with decreased LC3B1 to LC-3B2 conversion, as well as the increased P62 accumulation and decreased p-P62 expression. However, these effects were reversed by the RAC1 inhibitor EHT 1864. Similar results were observed for the silencing of S1P2 receptor in the cells, as shown by the decreased Beclin1 expression, decreased LC3B1 to LC-3B2 conversion, increased P62 accumulation, and decreased p-P62 expression. The smooth muscle activators were significantly decreased in the JTE-013 and EHT1864 groups, and the EHT 1864 + S1P2-SiRNA expression level was increased. S1P is involved in the progression of asthma and airway remodeling, which may be related to the activation of S1PR2 receptor and inhibition of autophagy through RAC1.

## Introduction

Sphingomyelin is a ubiquitous component of eukaryotic lipid bilayers, and sphingosine (N-acylsphingosine) is the backbone of sphingolipids, which is mainly generated by the ceramide synthesis and sphingolipid turnover. During the catabolism of sphingolipids, ceramide is deacylated to generate sphingosine. Subsequently, sphingosine would be phosphorylated by sphingosine kinase 1 and 2 (SphK1 and SphK 2) to form sphingosine-1-phosphate (S1P). S1P can be degraded either reversible by dephosphorylation and sphingosine acylation, or irreversibly by phosphohydrolase cleavage, to generate phosphoethanolamine and hexadecyl compounds. S1P contains five G-protein coupled receptor subtypes, termed S1P1-5, and most cells express one or more S1P receptor subtypes. S1P1 and SIP2 receptors can bind to S1P with high affinity, which is involved in many important cellular processes, including the cell proliferation, migration, and angiogenesis (Shi et al., 2019). Different S1P receptors have different coupling properties for the G protein family: S1P1 has coupling properties for the Gi/o family; while the S1P2 has coupling properties for the Gi/o, G12/13, and Gq protein families. These coupling properties allow direct regulation of small GTP enzymes, (i.e. the Rho, Rac, and Ras) (Sadok and Marshall, 2014). In addition, abundant S1P1 can be detected in endothelial cells, and S1P1 on the plasma membrane of aortic endothelial cells is negatively correlated with the abundance of inflammatory adhesion molecules. In adult mice, S1P has the ability to regulate the vascular development and microvascular barrier function (Galvani et al., 2015). Moreover, inhibition of S1P and its receptors blocks the allergic responses during pro-inflammatory responses in endothelial cells, and S1P2 receptor inhibition completely abolishes the TNF-α-induced expression of the vascular cell adhesion molecule 1 (VCAM-1) and intercellular adhesion molecule 1 (ICAM-1). Furthermore, inhibition of S1P binding to S1P2 directly inhibits the NF-κB pathway involved in the cytokine responses, and therefore inhibiting the pro-inflammatory response (Morel et al., 2016).

The mast cells (MC) have long been recognized as key effector cells in the allergic airway responses in asthma. After contacting with the sensitizing allergen, the mast cells would be activated by the cross-linked surface-bound IgE, leading to the degranulation and release of bioactive mediators (Zhang et al., 2013). It has been shown that S1P (as a sphingolipid metabolite produced by mast cells) could act as an important modulator of allergen-induced mast cell activation, which plays an important role in the pathogenesis of chronic asthma (Galli, 2017). S1P disrupts the integrity of respiratory epithelial cell barrier and activates the cholinergic receptors. Moreover, the elevated S1P levels have been observed in the bronchoalveolar lavage fluid (BALF) from asthmatic patients, which demonstrates the role of S1P in mast cell-dependent inflammatory and allergic responses. The SphK inhibitors attenuate the airway hyperresponsiveness and inflammation in a mast cell-dependent mouse model of allergic asthma (Price et al., 2013). In addition, S1P is a major modulator of the lymphocyte trafficking from the secondary lymphoid organs into the systemic circulation. The plasma S1P is mainly produced by the erythrocytes and vascular endothelial cells, while the lymphatic S1P is secreted by the lymphatic endothelial cells. The majority of plasma S1P is bound to high-density lipoproteins, which plays an important role in the immune homeostasis by regulating lymphopoiesis. Moreover, the S1P expression is required for the excretion of the B and T cells from the lymph nodes, and the mature and natural killer T cells from the thymus. It has been shown that, in the asthmatic mice, after treated with FTY-720 (a synthetic analog of S1P), the inhibition of Th2-related transcription factors could abolish the allergic inflammation and airway hyperresponsiveness induced by the acute allergen challenge (Bošnjak et al., 2019). On the other hand, in mice treated with SEW-2871 (an S1P1-selective agonist), significantly reduced TNF-α and IFN-γ levels would observed, therefore regulating the lymphocyte trafficking and development (Ni et al., 2015).

JTE-013, a potent and selective S1PR2 antagonist, could reverse the inhibitory effect of S1PR2 on vascular endothelial cells, which could also exert diverse effects by mediating multiple signaling pathways. S1PR2 has been shown to be able to regulate the ROCK1/Drp1 pathway to activate the destabilizing mediators in the endothelial barrier, and modulating the mitochondrial morphology and function by inducing HRGECs (Chen et al., 2019). It has been shown that S1P2 may prevent the bleomycin-induced acute inflammation and chronic fibrosis in mouse models (Park and Im, 2019b). However, JTE-013 could inhibit the asthmatic allergic responses by inhibiting the S1P2-mediated NF-κB activation and CCL3 production in bronchial epithelium. Intratracheal administration of JTE-013 may also be a promising treatment for bronchial asthma. S1P plays a key role in cardiomyocyte hypoxia through autophagy. It has been shown that, in the pulmonary fibrosis, the S1P lyase would act as an important modulator, involving the autophagy, and the S1P lyase over-expression attenuates the TGF-β- and S1P-induced differentiation of human lung fibroblasts by regulating the expression of LC3 and Beclin-1 (Huang et al., 2015). However, there are still no clear studies investigating whether S1P is involved in murine asthma through the autophagic responses. Therefore, in this study, the effects of S1P on asthma progression and the involvement of autophagy were investigated. Moreover, the related downstream pathways were also explored and studied.

## Materials and Methods

### Study Animals

Totally 30 male BALB/c mice, weighing 18 ± 5 g, were provided by the Laboratory Animal Center, Department of Medicine, Yanbian University (Yanji, Jilin, China). These animals were given a normal diet and subjected to adaptive housing condition for one week before following experiments. This study was approved by the Ethics Committee of Yanbian University.

### Animal Model Establishment and Grouping

**FIGURE 1:**
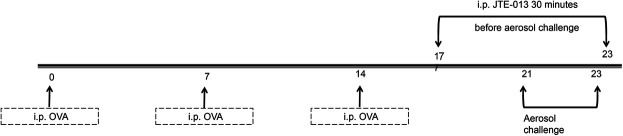
Establishment of asthma inflammation model. OVA was injected intraperitoneally for sensitization at days 0, 7, and 14. The asthma model was challenged by the OVA aerosol inhalation starting from day 21. The mice in the normal group were given with normal saline and aerosol inhalation. The mice in the JTE-013 group received the intraperitoneal injection of JTE-013, beginning on day 17. The mice in the normal group were injected with the saline, at 30 min before nebulization.

For the establishment of asthma mouse model, totally 30 BALB/C mice were randomly divided into the normal control, model and treatment groups (n = 10 per group), respectively. Except for the normal control group, the mice from the model and treatment groups were intraperitoneally injected with 0.05 g aluminum hydroxide suspension (OVA; Sigma, St Louis, MO, United States) and 0.56 ml Al(OH)_3_ dissolved in 200 μL saline on days 0, 7, and 14. Starting from day 21, the animal models were treated with 0.1 g OVA dissolved in 10 ml saline for challenging, for three consecutive days. Starting from day 17, the drug was administered for seven consecutive days, through intraperitoneal injection with 8 mg/kg JTE-013 (Sigma) in 200 µL saline (on days 21–23, the drug was given at 30 min before challenging) (Figure 1).

Forty BALB/C mice were randomly divided into the normal, model, JTE-013 and RAC1 inhibitor groups, respectively. For the RAC1 inhibitor group, the mice received a tail vein injection of RAC1 inhibitor (3 mg/kg) over days 0–20.

**FIGURE 2:**
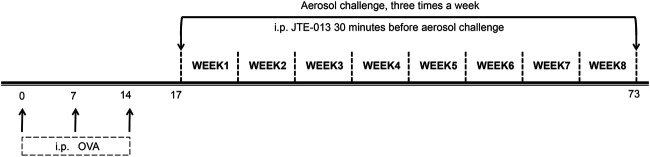
Establishment of airway remodeling model in chronic asthma. The OVA sensitization was performed by intraperitoneal injection at days 0, 7, and 14. The OVA was nebulized, three times a week, for eight consecutive weeks, starting from day 17. JTE-013 was intraperitoneally injected at 30 min before aerosol inhalation in the JTE-013 group, and EHT1864 was intraperitoneally injected at 30 min before JTE-013 administration in the EHT1864 group, at 6–8 weeks.

On the other hand, the mouse model of chronic asthma was established to examine the role of autophagy. On days 1, 7 and 14, respectively, each mouse in the control group was intraperitoneally injected with 200 μL saline, while the mice from the model and treatment groups were intraperitoneally injected with sensitization suspension (10 μg OVA +1 mg aluminum hydroxide +200 μL saline), starting from day 17. The mice from the model and treatment groups were nebulized with challenge solution (0.1 g OVA +10 ml saline, i.e., 1% OVA in normal saline) for 30 min, three times per week, for eight consecutive weeks. For the model group, JTE-013 was given intraperitoneally at 8 mg/kg, daily starting from day 17. For the control and model groups, the mice were given with intraperitoneal injection of saline, at 30 min before challenging (Figure 2).

### Study Cell Lines and Cell Culture

Type II alveolar epithelial cells MLE12 and rat airway smooth muscle cells were purchased from the Fuheng Biological Technology Co., Ltd., Shanghai, China. The MLE12 cells were cultured in the DMEM/F12 complete medium, containing 10% FBS, in a 37 °C, 5% CO_2_ incubator. The airway smooth muscle cells were cultured in the DMEM complete medium, containing 10% FBS, in a 37 °C, 5% CO_2_ incubator.

### Airway Reactivity Assessment

At 24 h after the last challenging, the airway hyperresponsiveness was assessed by the lung resistance measurement (SCIREQ, Montreal, Canada). Briefly, mice were anesthetized with pentobarbital sodium (100 mg/kg) through intraperitoneal injection. The trachea was exposed, and the cannula was inserted. The mice were treated with methacholine aerosol at indicated doses, i.e., 3.125, 6.25, 12.5, 25, and 50 mg/ml, respectively. Then the airway hyperresponsiveness was assessed, and the lung resistance was quantified accordingly.

### Serum Sample Preparation

At 24 h after the last challenging, the mice were anesthetized with ether, and the blood sample was collected through orbit (1 ml from each mouse). The blood sample was kept at room temperature for 3 h, followed by the centrifugation at 1,500 r/min at 4 °C for 15 min. The supernatant was obtained, and the IgE content in the serum was measured using the ELISA kits, according to the manufacturer’s instructions.

### Measurement of Inflammatory Factors in Tissues

Lung lavage was performed with 1 ml saline, and the bronchoalveolar lavage fluid was obtained (approximately 0.8 ml). After centrifugation at 1,500 r/min at 4 °C for 5 min, the supernatant was obtained. Totally 100 μL sample was added into the 96-well plate containing the primary antibodies (IL-1β, IL-4, and IL-5; all from Cell Signaling, Beverly, MA, United States), which was gently shaken and then placed in a water bath at 37 °C for 2 h. The solution was discarded, and the biotin-labeled antibody working solution was added to incubate the plate for 1 h. After washing for 3 times (soaking for 2 min each time), 100 μL horseradish peroxidase-labeled avidin working solution was added, and the plate was heated for 1 h. Then the liquid was discarded, and 90 μL substrate solution was added into each well, which was kept in dark for 15 min. The reaction was stopped by adding 50 μL stop solution. The absorbance at 450 nm was detected within 5 min, and the values were calculated.

### Classification of Cells in Bronchoalveolar Lavage Fluid

The cells were collected in 900 μL saline, which were centrifuged at 1,000 r/min for 10 min and at 1,500 r/min for 5 min. The cell smear was obtained after naturally drying, which was subjected to the Swiss Giemsa staining for 3 min. Then the equal amount of PBS was added, and the staining solution and residue on the slide was washed out with water. After naturally drying, the slide was then covered. The number and type of cells were observed and counted under microscope.

### Measurement of Inflammatory Factors in Cells

The alveolar epithelial BLE12 cells were seeded onto the 6-well plates, at the density of 3×10^4^ cells/cm^2^ which were incubated at 37 °C for 24 h. For the treatment group, 200 μL JTE-013 (2 mmol/L in ethanol) was added into each well. Except for the blank control, 200 μL LPS (with a final concentration of 2000 ng/ml) was added to each well, which was then cultured for 24 h. Then the cells were centrifuged at 1,000 r/min at 4 °C, and the supernatant was collected. According to the instructions of the ELISA kit, 100 μL saline was added to each well, which was cultured at 37 °C for 2 h. The biotin-labeled antibody working solution and the horseradish peroxidase-labeled avidin working solution were added, followed by the addition of the substrate and stop solution. Therefore, the contents of IL-1, IL-4 and IL-5 in the supernatant were measured.

### Assessment of Airway Hyperresponsiveness

Asthma was measured on day 2 after the last OVA challenge. Mice were placed in a pneumatic plethysmography chamber, and given with increasing concentrations of acetylcholine (2.5–50.0 mg/ml) by nebulization. The airway responses were assessed every 3 min. The breathing curve was obtained, and the airway resistance (expressed as the Penh measurements) was calculated.

### Lung Histopathological Treatment

After the bronchoalveolar lavage fluid collection, the left lung was removed and fixed in 4% formalin overnight. The tissue was embedded in paraffin, and cut into 4 μm sections. Then the sections were subjected to the AB-PAS staining (Solaibao, Beijing, China), and the pathological changes of lung tissue and the inflammatory cell infiltration were observed under light microscope. In addition to the AB-PAS and HE staining, the tissue sections were processed for the Masson staining of the airway remodeling model.

### Western Blot Analysis

Cells or tissues were harvested and lysed with lysis. The lysate was kept on ice for 30 min, followed by the centrifugation at 12,000 rpm at 4 °C for 20 min. The protein concentration was measured by the BCA assay. The protein samples were separated by 10% SDS-PAGE, and then electronically transferred onto the PVDF membrane. After blocking with 5% non-fat milk at room temperature for 1.5 h, the membrane was incubated with the primary antibody (RAC-1, 1 : 1,500 dilution; JNK, 1 : 1,000 dilution; Beclin-1, 1 : 1,000 dilution; and LC-3B, 1 : 1,000 dilution; all from Cell signaling), at 4 °C overnight. After washing, the membrane was incubated with the secondary antibody (1 : 2000 dilution) at room temperature for 1 h. After the exposure development, the protein bands were imaged and analyzed.

### Immunohistochemistry

The paraffin-embedded sections were routinely deparaffinized and washed with PBS. Then the sections were placed in 0.01 M citrate buffer (pH 6.0) in a microwave oven and cooled to room temperature. After washing, the endogenous peroxidase was removed by dropping with 3% H_2_O_2_, and the sections were subjected to blocking for 10 min. The section was incubated with primary antibody (Rac 1, Beclin-1, LC-3B, and P62; all in 1:200 dilution; all from cell signaling), at 4 °C overnight. The sections were then incubated with biotinylated secondary antibodies at 37 °C for 30 min, followed by the treatment with HRP-labeled avidin at 37 °C for 20 min. The DAB chromogen solution was added dropwise for approximately 3 min, followed by extensive tapping with tap water for 10 min. Then the sections were observed under microscope.

### Immunofluorescence

The cells were washed and fixed with formaldehyde for 30 min. After washing, the cells were permeabilized with 0.5% Trition at room temperature for 30 min. The supernatant was discarded, and the cells were subjected to blocking with 5% BSA for 1 h. The cells were incubated with corresponding primary antibody at 4 °C overnight. Then the cells were incubated with the secondary antibody (1 : 200 dilution) in dark at 4 °C for 2 h. After counterstained with DAPI (20 μL/well), the cells were observed under fluorescence microscope.

### Statistical Analysis

Data were expressed as mean ± SD. Statistical analysis was performed using the Graph Pad Prism software. One-way analysis of variance followed by SNK method was used for comparisons among groups. *p* < 0.05 was considered as statistically significant.

## Results

### JTE-013 Inhibits Inflammation in OVA-Induced Asthmatic Mice

**FIGURE 3:**
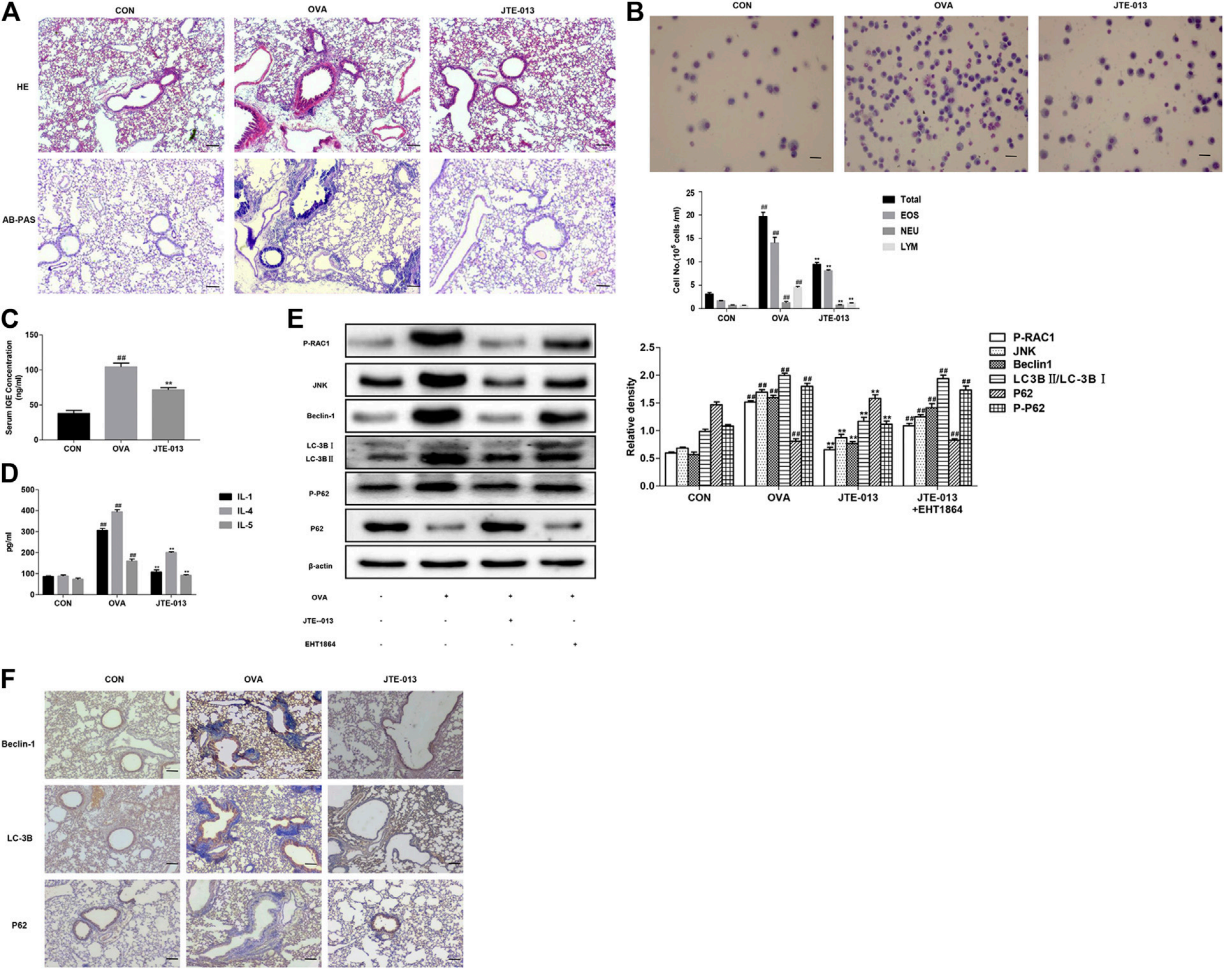
JTE-013 inhibits inflammatory responses in asthma by inhibiting autophagy **(A)** Inflammatory cell infiltration in the lung tissue was analyzed by the HE staining. The goblet cell proliferation was evaluated by the AB-PAS staining **(B)** Wright-Giemsa staining for counting of cells in the bronchoalveolar lavage fluid. EOS, eosinophils; NEU, neutrophils; and LYM, lymphocytes **(C)** Measurement of serum IGE in asthmatic mice **(D)** Content of inflammatory cytokines IL-1, IL-4, IL-5 in the bronchoalveolar lavage fluid **(E)** Effect of EHT1864 on inflammation and autophagy in asthma **(F)** Immunohistochemical analysis of tissue expression levels of Beclin-1, LC-3B and P62. Scale bar, 50 μm. Compared with the control, ^#^ *p* < 0.05, ^##^ *p* < 0.01; and compared with the OVA-treatment group, *p < 0.05, ***p < 0.01.

The histopathological changes in the lungs were detected with the HE and AB-PAS staining. Our results showed significant inflammatory cell infiltration, airway wall thickening, and airway smooth muscle thickening in the periphery of the trachea of asthmatic mice, compared with the normal group. Moreover, the JTE-013 treatment significantly reduced the pathological changes in asthma. The results from the AB-PAS analysis showed that, compared with the normal group, the goblet cell hyperplasia was evident and the inflammatory cells were increased. Moreover, the JTE-013 treatment significantly improved the pathological changes of asthma (Figure 3A).

Based on the findings that JTE-013 is more effective when administered during the challenge in an asthma model (Park and Im, 2019a), in this study, JTE-013 was intraperitoneally injected at 30 min before challenging. Our results showed that, the numbers of total cells, eosinophils, basophils, and neutrophils in the bronchoalveolar lavage fluid were significantly increased after the OVA-induced asthma. Moreover, the contents of these inflammatory cells were significantly decreased by the JTE-013 treatment (Figure 3B). These results suggest that JTE-013 may play an inhibitory role in the asthmatic inflammation. When asthma develops, the B cells endocytose the processed antigens and release the inflammatory cytokines (such as IL-4 and IL-5), which further promotes the B cell activation and subsequently produce the specific IgE antibodies to cross-link with mast cell eosinophils to release the inflammatory mediators. Moreover, the mast cells, eosinophils, neutrophils, epithelial cells, macrophages, and endothelial cells could produce the inflammatory mediators. As shown in Figure 3B, differential counts of these inflammatory cells were found in the bronchoalveolar lavage fluid. The serum IgE and the levels of IL-1, IL-4, and IL-5 in the bronchoalveolar lavage fluid, were measured with ELISA. Our results showed that, compared with the normal control group, the contents of these factors were significantly increased in the asthma model group. Moreover, the contents of IgE (Figure 3C), and the levels of IL-1, IL-4, and IL-5, were significantly down-regulated in the models after the JTE-013 administration (Figure 3D). Taken together, our results suggest that, JTE-013 could inhibit the inflammation in OVA-induced asthmatic mice.

### JTE-013 Inhibits Autophagy Through RAC1/JNK and Reduces Autophagy-Related Protein Expression

Beclin1, LC3 and P62 are autophagy-related proteins. Therefore, the expression levels of Beclin-1, LC-3B, and P62 were detected by the Western blot analysis. Our results showed that the expression level of Beclin-1 was increased in the lung tissue of the OVA-induced mice, but not in the lung tissue of the JTE-013-treated mice. On the other hand, the transformation of LC-3B was enhanced and the sequel of P62 was attenuated in the OVA-induced asthmatic mice, which was reversed by the JTE-013 treatment. Interestingly, the phosphorylation level of P62 was also reduced after the JTE-013 treatment, suggesting that JTE-013 inhibited the autophagic process. To examine the effect of S1P on RAC1 in the airways, RAC1 expression in lung pathological tissue sections was examined using immunohistochemistry, which showed that JTE-013 significantly reduced the positive expression of RAC1 in lung tissues during the inflammatory phase of asthma (Supplementary Figure S1A) and airway remodeling (Supplementary Figure S1B). Moreover, the phosphorylation levels of RAC-1 and JNK were further examined herein. Our results showed thatJTE-013 reduced the phosphorylation levels of RAC-1 and JNK in the lung tissues of the OVA-induced mice. The mice were treated with the RAC-1 inhibitor. Our results showed that the RAC-1 inhibitor attenuated the therapeutic effect of JTE-013 on the expression of autophagy-related proteins in the lung tissue of asthmatic mice. These results demonstrate that S1P2 can regulate autophagy through the S1P2/RAC-1/JNK pathway, which plays a therapeutic role in the asthmatic mice (Figure 3E). These results suggest that JTE-013 inhibits the autophagy through the RAC1/JNK, and reduces the expression levels of the autophagy-related proteins.

### S1P2 Receptor Antagonist Inhibits Autophagy-Related Protein Expression

Effects of the S1P2 receptor antagonist on the expression levels of autophagy-related proteins were investigated. Our results showed that, the expression levels of Beclin-1, LC-3B, and P62 in the airway epithelium were significantly increased in the model group, while the Beclin-1 and LC-3B expression levels were significantly decreased after the intraperitoneal injection of the S1PR2 antagonist (JTE-013). The accumulation of P62 was increased, compared with the model group (Figure 3F). These results suggest that the S1P2 receptor antagonist could inhibit the expression levels of the autophagy-related proteins.

### JTE-013 Attenuated LPS-Induced Inflammation Produced by Alveolar Epithelial Cells and Inhibited Autophagy via RAC1/JNK

**FIGURE 4:**
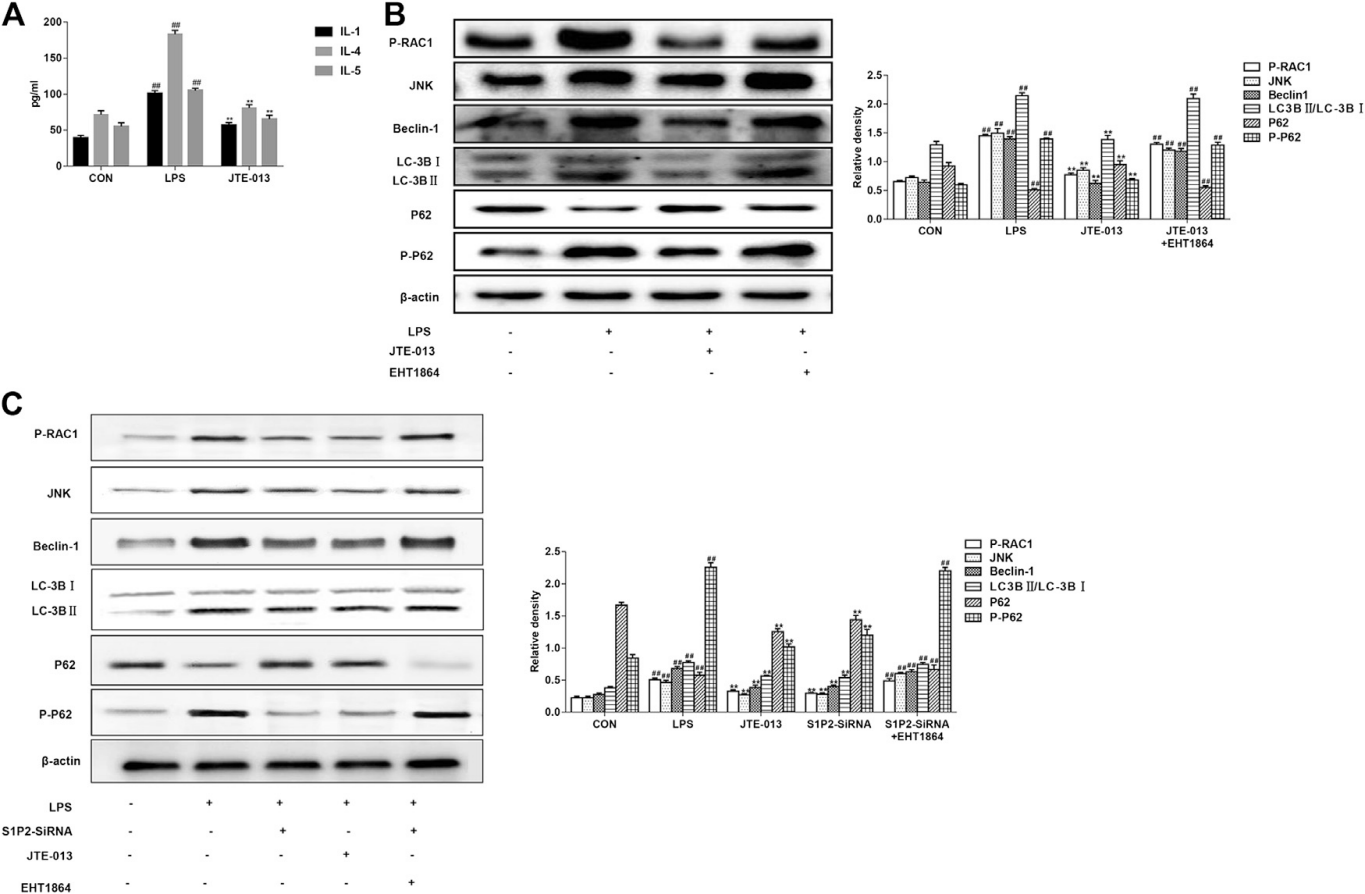
JTE-013 suppresses inflammatory responses in asthma by inhibiting autophagy in epithelial cells **(A)** Content of inflammatory cytokines IL-1, IL-4, IL-5 in cell culture supernatant **(B)** Western blot analysis of protein expression levels of P-RAC1 and JNK, as well as the autophagy-related proteins Beclin-1, p62, P-p62 and LC-3B transformation **(C)** Effect of S1P2 receptor silencing on inflammation and autophagy in LPS-induced alveolar epithelial cell inflammation. Compared with the untreated cells, ^#^ *p* <0.05, ^##^ *p* < 0.01; compared with the LPS-induced inflammation, *p < 0.05, **p < 0.01.

It is now well-established that LPS can induce inflammatory responses, which stimulate and increase S1P and promotes the binding of S1P to its receptor. Therefore, the inflammatory factors in the cell culture supernatant were measured herein. Our results showed that, JTE-013 reduced the levels of pro-inflammatory cytokines (IL-1) and inflammatory cytokines (IL-4 and IL-5) produced by the alveolar epithelial cells (Figure 4A), suggesting that JTE-013 has the effect of inhibiting inflammation, which may be related to the inhibited inflammation in asthma. Moreover, the protein contents were quantified with the BCA method. As shown in Figure 4B, JTE-013 significantly reduced the LPS-induced increased RAC1/JNK levels, and the inhibition would be reversed by the RAC1 inhibitor treatment (Figure 4C). Moreover, the contents of autophagy-related proteins (Beclin-1 and LC-3B) were consistent with RAC1/JNK. On the contrary, the P62 accumulation was decreased in the model group, and the P62 content was significantly increased after the JTE-013 treatment. These results suggest that, JTE-013 could attenuate the LPS-induced inflammation produced by alveolar epithelial cells and inhibit the autophagy via RAC1/JNK.

### JTE-013 Inhibited OVA-Induced Airway Remodeling

**FIGURE 5:**
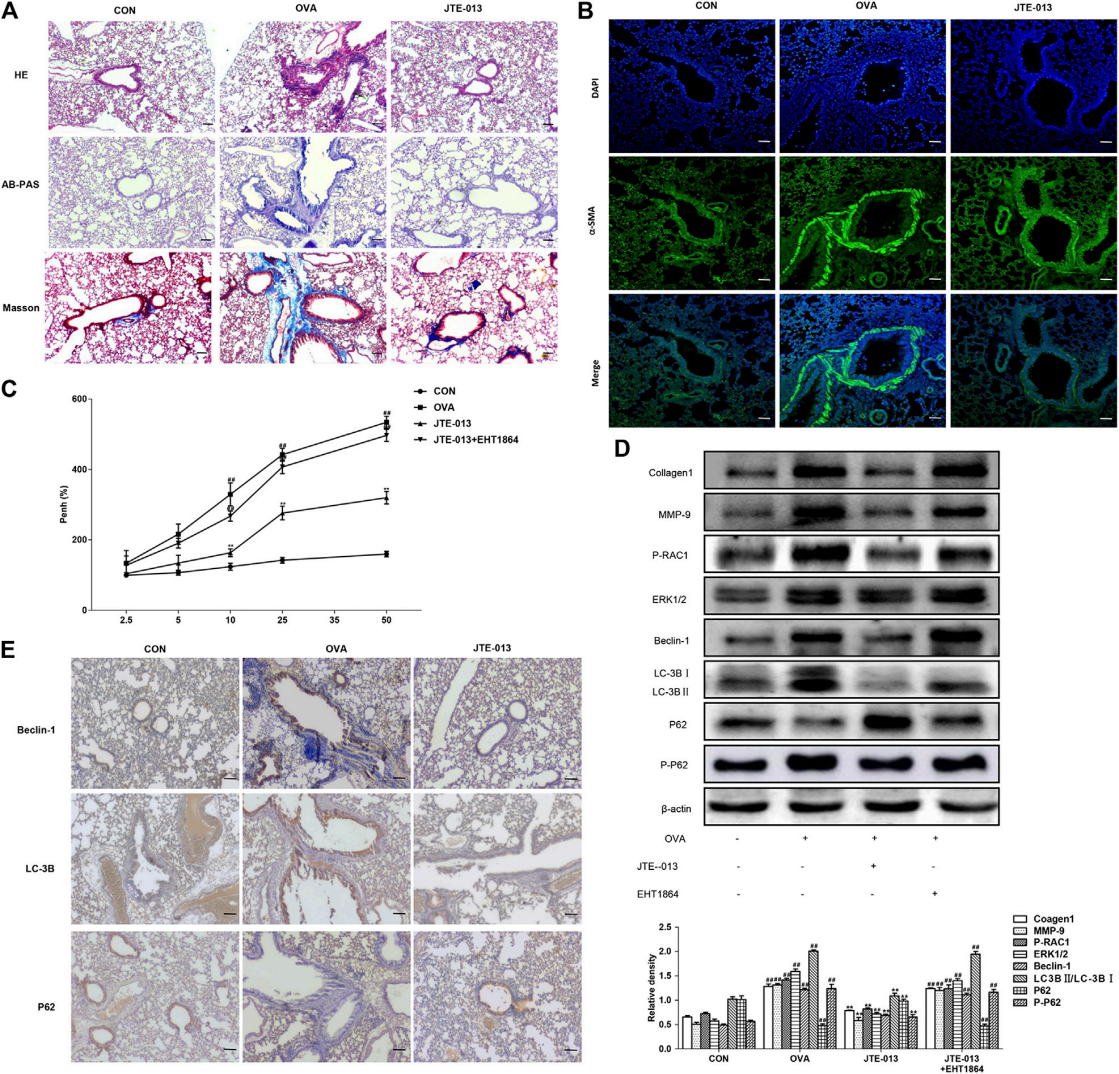
JTE-013 inhibits airway remodeling in asthma by inhibiting autophagy in animal models **(A)** Inflammatory cell infiltration in the lung tissue was analyzed by the HE staining. The goblet cell proliferation was evaluated by the AB-PAS staining. The collagen deposition outside the airway was assessed by the Masson method **(B)** Detection of α-SMA expression in the airway smooth muscle by immunofluorescence **(C)** Assessment of Airway Hyperresponsiveness. Asthma was measured on day 2 of the last OVA challenge **(D)** Western blot analysis of protein expression levels of P-RAC1, ERK1/2, MMP9, Collagen1 and the autophagy-related proteins Beclin-1, p62, P-p62 and LC-3B transformation **(E)** Immunohistochemical analysis of tissue expression levels of Beclin-1, LC-3B and P62. Scale bar, 50 μm. Compared with the control, ^#^ *p* < 0.05, ^##^ *p* < 0.01; and compared with the OVA-treatment group, *p < 0.05, **p < 0.01.

The airways are chronically stimulated by the inflammation and the tissues are repeatedly damaged, further resulting in the thickened airway smooth muscle and accumulated inflammatory cells. Our results from the HE staining showed that, compared with the normal group, the inflammatory cell infiltration was significantly increased in the OVA-induced airway remodeling model, which could be reduced by the JTE-013 treatment. Meanwhile, our results from the AB-PAS analysis showed that the JTE-013 treatment decreased the proliferation of goblet cells on the trachea. The Masson results showed that the collagen deposition was significant in the model group compared with the control group, which could be significantly improved by the JTE-013 treatment (Figure 5A).

The actin content has great influence on the contraction ability of smooth muscle, which can reflect the change of smooth muscle quantity and contraction ability. Our results from immunofluorescence showed that the contents of a-SMA in airway smooth muscle in the model group were significantly increased compared with the normal group, which could be inhibited by the JTE-013 treatment (Figure 5B).

The lung resistance was measured on day 2 after the last challenge. Our results showed that the lung resistance was significantly higher in mice inhaling 10, 25, and 50 mg/ml methacholine, compared with the normal group (*p* < 0.01). Moreover, the intraperitoneal injection of JTE-013 significantly decreased the methacholine (10, 25, and 50 mg/ml)-induced lung resistance (*p* < 0.01) (Figure 5C). This decreasing trend could be significantly counteracted by the treatment of the RAC1 inhibitor, which significantly increased the pulmonary resistance in the EHT1864 group compared with the normal group (*p* < 0.01). These results suggest that the administration of JTE-013 reduces the OVA-induced airway remodeling in the mouse asthma model, which could be counteracted by the RAC1 inhibition.

The expression levels of autophagy-related proteins were further investigated. Our results showed that, along with the progression of OVA-induced airway remodeling, the expression levels of autophagy-related proteins were decreased after treatment with the S1P2 receptor antagonist JTE-013, while the P62 accumulation would be reduced in airway remodeling by JTE-013. Moreover, the inhibition of RAC1 significantly inhibited the expression levels of airway remodeling-related proteins and ERK1/2. Furthermore, the JTE-013 treatment reduced the OVA-induced airway remodeling, which could be counteracted by the RAC1 inhibitor (Figure 5D). These results suggest that, the JTE-013 could inhibit the OVA-induced airway remodeling.

### JTE-013 Attenuated Autophagy in Airway Remodeling

In line with the asthma results, the expression levels of the autophagy-related proteins (Beclin-1 and LC-3B) were increased with the progression of OVA-induced airway remodeling. Moreover, the expression levels of autophagy-related proteins were decreased after the treatment with the S1P2 receptor antagonist JTE-013, in contrast to the decreased P62 accumulation in the airway remodeling, which could be counteracted by JTE-013 (Figure 5E). These results suggest that, JTE-013 could attenuate the autophagy in the airway remodeling.

### Effects of JTE-013 Inhibition in Airway Smooth Muscle Cells

**FIGURE 6:**
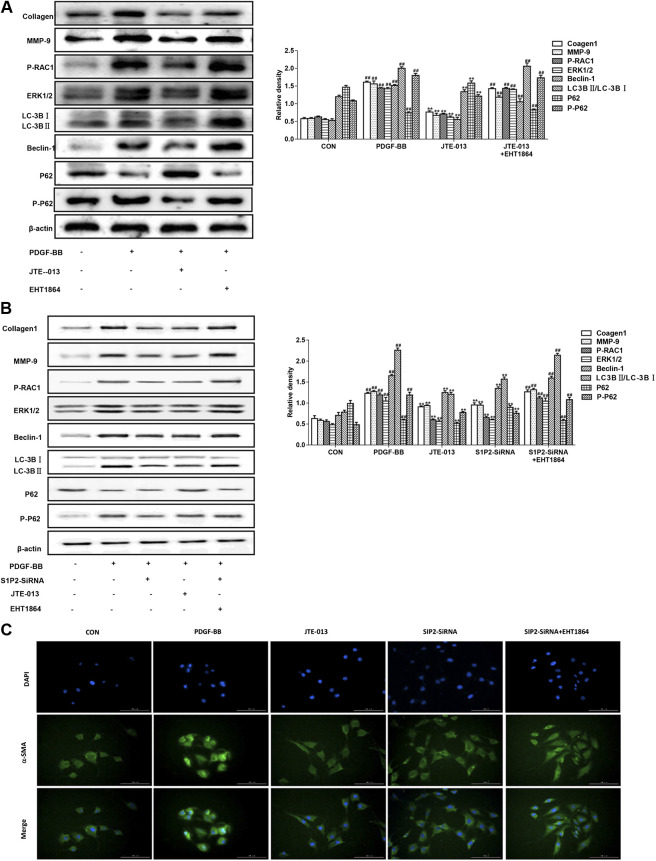
Inhibition of JTE-013 in airway smooth muscle. The EHT1864-treated cells were treated at 30 min before the treatment of JTE-013. The measurement was taken after 24 h **(A)** Western blot analysis of protein expression levels of P-RAC1, ERK1/2, Collagen-1, MMP-9 and the autophagy-related proteins Beclin-1, p62, P-p62 and LC-3B transformation **(B)** Effect of S1P2 receptor silencing on PDGF-BB-induced autophagy in the airway smooth muscle cells **(C)** Immunofluorescence expression levels of α-smooth muscle actin (α-SMA, green) in smooth muscle cells. DAPI, blue. Scale bar, 50 μm. Compared with the untreated cells, ^#^ *p* < 0.05, ^##^ *p* < 0.01; and compared with the PDGF-BB-induced airway smooth muscle cells, *p < 0.05, **p < 0.01.

The PDGF-BB induced the airway smooth muscle cell proliferation and induced the airway remodeling model. As shown in Figure 6A, in consistent with the LPS-induced inflammation model, the JTE-013 treatment significantly reduced the PDGF-BB-increase expression levels of RAC1/ERK1/2, as well as the Beclin-1 and LC-3B. However, the inhibiting effects could be inhibited by the RAC1 inhibitor. Moreover, the P62 accumulation in the model group was reduced, and the P62 content was significantly increased after the JTE-013 treatment.

### Silencing S1P2 Receptor Inhibited Asthma Inflammation and Airway Remodeling

In the LPS-induced asthma inflammation model, the effects of S1P2-SiRNA were similar to the JTE-013. Our results showed that, the expression levels of S1P2-SiRNA histone were decreased, indicating that S1P2 was involved in the development of asthma inflammation following the treatment with the RAC1 inhibitor EHT 1864. The downward trends in the protein expression levels would be reversed, further suggesting that S1P2 can participate in the occurrence of asthma disease through the RAC1 pathway (Figure 4C). Moreover, consistent results were observed in the PDGF-BB-induced airway remodeling model, i.e., the S1P2Si-RNA reduced the expression levels of Collagen-1 and autophagy-related proteins, which was induced by the RAC1 inhibitor EHT 1864. Furthermore, the results from the immunofluorescence showed that the expression levels of smooth muscle agonists were decreased in the S1P2-SiRNA group, while the expression levels of airway smooth muscle actin were significantly increased in the EHT1864 group compared with the normal group (Figure 6B). These results would be verified by the detection of the smooth muscle actin expression levels in the airway smooth muscle cells based on the immunofluorescence (Figure 6C). Taken together, these results suggest that, S1P2 receptor silencing inhibits the asthma inflammation and the airway remodeling.

## Discussion

Asthma is one of the common chronic diseases, with about 300 million asthmatics cases throughout the world. The clinical manifestations of asthma mainly include the recurrent wheezing, breathing shortness and coughing. Moreover, asthma is a chronic airway inflammatory disease, which involves a variety of cells and cellular components. The main features of asthma include the chronic airway inflammation, eosinophilic inflammation and increased goblet cell mucus secretion, and long-term inflammatory secretion, which might lead to the airway smooth muscle thickening and airway hyperresponsiveness.

S1P is the main regulator of lymphocytes from the secondary lymphoid organs into the systemic circulation, which plays an important role in the immune homeostasis. The expression of S1P is necessary for the T cell maturation, and the CD^4+^ cells also play important roles in the immune regulation and defense. The T helper cells can be divided into the Th1 and Th2 cells based on the secretion of different cytokines, and the Th1/Th2 cytokine balance is important for the maintenance of the normal body immunity. However, the Th1/Th2-mediated immune imbalance is the main mechanism of the airway inflammation in asthma, which is normally dominated by the Th1-type cytokines, while the Th2-type cytokine secretion would be increased in patients with asthma. In this study, our results showed that, the inflammatory cytokines (Th2 type) would be detected in the bronchoalveolar lavage fluid, and the JTE-013 treatment significantly reduced the contents of IL-1, IL-4, and IL-5 in the bronchoalveolar lavage fluid. It has been shown that S1P is a sphingolipid metabolite produced by the mast cells, which is an important modulator for the activation of mast cells. Specific SphK inhibitors could attenuate the airway inflammatory responses in a mast cell-dependent mouse model of allergic asthma. In the models, the mast cells are activated by the cross-linking surface-bound IgE after exposure to allergens, which lead to subsequent degranulation and release of activating mediators (Galli, 2017). Antigen-stimulated mast cells can release S1P into the interstitium, thus regulating the inflammatory processes. Therefore, our results showed that, the serum IgE expression level was enhanced in the asthmatic mice, which could be inhibited by the JTE-013 treatment, indicating that S1P inhibited the mast cell activity and reduced the inflammation in asthma through S1PR2. Moreover, the HE and AB-PAS staining showed that, the JTE-013 treatment significantly reduced the inflammatory cell infiltration around the airways and reduced the goblet cell mucus production. IL-13 has been reported to be able to promote the formation of goblet cells, thereby activating the autophagy to induce mucus secretion by epithelial cells. Moreover, IL-13 could prolong the stimulation of epithelial cells, thus elevating the expression levels of autophagy-related protein LC3. Furthermore, the blocking of autophagy can decrease the asthma production by decreasing the reactive oxygen species generation (Dickinson et al., 2016). Similarly, we found that the S1PR2 antagonist JTE-013 not only inhibited airway inflammation and wall thickening (Supplementary Figure S1), but also decreased the expression of RAC1. RAC1 play an important signaling role in proliferation and differentiation as well as cellular transcription factor regulation. We suggest that S1P can activate RAC1 via receptor two and activate autophagy signaling.

LPS can activate S1P production and activate its downstream S1P1-5 related receptors and their downstream pathways (Du et al., 2012; Imeri et al., 2015; Jin et al., 2018). In airway inflammation, S1P also functions in the airway smooth muscle during the airway remodeling. S1P has been proposed to affect the airway remodeling by stimulating the proliferation of human airway smooth muscle cells. S1P would possibly inhibit the myosin phosphatase via inhibiting the RhoA (Sun et al., 2018). The SPHK activation and the intracellular calcium release have also been shown to be involved in the contraction of asthmatic airways, as well as the muscarinic receptors. Moreover, the systemic administration of S1P would elevate the airway resistance and cholinergic activity in the mouse whole-lung model. Furthermore, based on the microarray analysis, no less than 88 genes regulated by S1P have been identified in the smooth muscle, including the genes involved in the cell proliferation and airway remodeling (such as HBEGF, RGS4, and PLAUR). Three of the five receptors for S1P that have been detected in the airway smooth muscle are expressed in smooth muscle cells, (i.e., the S1P1-3). Moreover, S1PR2 and S1PR3 are essential for the airway smooth muscle, which may be activated through the Rho-associated kinase pathway (Fuerst et al., 2014). In this study, PDGF-BB was used to induce the proliferation of rat airway smooth muscle cells. The JTE-013 was tested for the cell proliferation and inhibition. Our results showed that JTE-013 inhibited the cell proliferation at the dose of 25 ng/ml. PDGF-BB could promote the proliferation of airway smooth muscle cells, and the Sphk1/S1P signaling regulates the PDGDF-BB-stimulated pulmonary artery smooth muscle cell proliferation (Li et al., 2018). The cellular proliferation would be inhibited by the S1PR2 antagonist JTE-013. The expression levels of the cellular signature proteins Collagen1A and MMP-9 were further examined. SMA-α could be used as the marker for airway smooth muscle to reflect the smooth muscle number and contractile capacity. Studies have shown that the effect of PDGF on S1P can play a role in airway smooth muscle cells by forming a complex with S1P receptors through its receptor (Wang et al., 2019) and promoting mitotic signaling (Waters et al., 2003). It has been shown that impaired mouse embryonic fibroblasts in PDGF-induced migration is thought to be caused by S1P deficiency (Kondo et al., 2014). Therefore, considering the effect of PDGF on S1P, we selected different stimulators for cells. In airway smooth muscle cells, we additionally compared the effects of S1P and PDGF on a-SMA expression, and found that S1P and PDGF induced similar trends on a-SMA expression (Supplementary Figure S2). Our results from the immunofluorescence showed that PDGF-BB increased the expression levels of SMA-α, which could be inhibited by JTE-013. Repeated inflammatory stimuli would lead to the airway hyperresponsiveness and airway remodeling. In this study, the OVA-induced chronic airway remodeling model in mice was used, and our results showed that the JTE-013 treatment significantly reduced the inflammatory cell infiltration induced by OVA, decreased the airway smooth muscle thickening, reduced the goblet cell mucus production, and declined the extra-airway collagen deposition. Moreover, the JTE-013 treatment had a therapeutic effect in the model of chronic airway remodeling.

MMP-9 could by produced not only by the inflammatory cells, but also by the lung tissue cells and bronchial epithelial cells in acute asthma (Naik et al., 2017). Expression levels of SMAD2/3 and Collagen1A were associated with the airway hyperresponsiveness in asthma, and the JTE-013 administration significantly decreased the airway hyperresponsiveness-related proteins in asthma, indicating that the S1P2 receptor is involved in both inflammation and airway remodeling processes in asthma.

Autophagy can contribute to the maintenance of the cellular homeostasis and the cell survival. Under physiological conditions, autophagy requires the targeting of adaptors to degrade the mis-folded proteins and dysfunctional organelles, thus regulating the organelle homeostasis, including the mitochondria and endoplasmic reticulum. On the other hand, under stress conditions (such as starvation and hypoxia), the AMPK activation, inhibition of mTORC1, activation of autophagy, and non-specific degradation of damaged cellular components or organelles contribute to the maintenance of the normal cellular function and activities. When autophagy is activated, the autophagosomes would be formed in the cytoplasm, which encapsulate the praise components needed to be degraded, and then the autophagosomes move toward and bind to lysosomes, forming the autophagy-lysosomes, where the materials encapsulated are subsequently degraded. Previous studies have shown that ATG5 is involved in the autophagosome formation, which would form complex with ATG12 and ATG16L1, in patients with refractory asthma (Karunakaran et al., 2019). Moreover, the formation and activation of the autophagy-specific ATG14-Beclin1-p150-PI3-Kinase complex, and the autophagy initiation and development, would be promoted during asthma, during which the T cells are activated and the cytokine secretion is affected by the B cells. The presence of autophagosomes in the T cells is related to the high expression of membrane protein LC-3B (Poon et al., 2012). Furthermore, the blocking of S1PR2 would inhibit the IL-6 secretion and the autophagic process of microglia, suggesting that S1P1 mediates the S1P-induced inflammation and increase in autophagy through S1PR2 (Nedjic et al., 2008). The expression levels of Beclin-1 and LC3B in the airway epithelial cells of acute inflammation model and chronic airway remodeling model were higher than the normal control group. In addition to Beclin-1 and LC-3, the autophagic flux status has always been judged by the detection of soluble p62 protein, insoluble p62 protein, and LC3B-1/LC3B-2 conversion. Reduced conversion from LC3BⅠ to LC3BⅡ by the soluble p62 indicates the activation of autophagic flux, autophagic process, and the related downstream pathways. On the one hand, p62 can directly act on the raptor proteins to activate the mTORC1 complex and negatively regulate the autophagic activity (Wang et al., 2014). Moreover, the autophagy inhibition would lead to the p62 aggregation and the subsequently increased mTORC1 levels, followed by the autophagy inhibition, furthermore promoting the p62 aggregation. Our results showed that, the p62 accumulation in the mouse lung tissue was reduced after the JTE-013 treatment, indicating activated autophagy in asthma. The activation was blocked by the inhibition of the S1PR2 receptor. These results suggest that JTE-013 could reduce the pathological changes of OVA in mice by inhibiting autophagy. S1P has been shown to be able to activate the signaling molecule GTPase Rac via the S1PR1 receptor, inducing the muscle protein translocation and promoting cell rearrangement. S1PR1 activates the RAC1 and initiates the endothelial cell barrier protecting function (Rutherford et al., 2013).

To explore the relationship between S1PR2 and RAC1, the RAC1 inhibitors were used in both cells and animals. The RAC1 inhibitors could abrogate the therapeutic effects of JTE-013 in asthma. The PI3K/Akt and JNK pathways can inhibit the mTOR pathway and the autophagic process (Cheng et al., 2019). Activation of ERK1/2 is critical for the signal transduction from surface receptors to nucleus, involving GTPase two Ras, Raf21, serine/threonine kinases, and MEK dual-specific kinases. The Ras/Raf/MEK/ERK signaling pathway is a cascade of small GTP-linked activated receptor tyrosine kinases and cytoplasmic proteins. RAC1 can activate the MAPK family p38, and ERK pathways. In this study, the JNK and ERK1/2 from the MAPK family were observed. Our results showed that, the expression levels after the treatment of RAC1 inhibitor were consistent with the expression levels of autophagy-related proteins, suggesting that S1PR2 exerted autophagy through RAC1.

**FIGURE 7:**
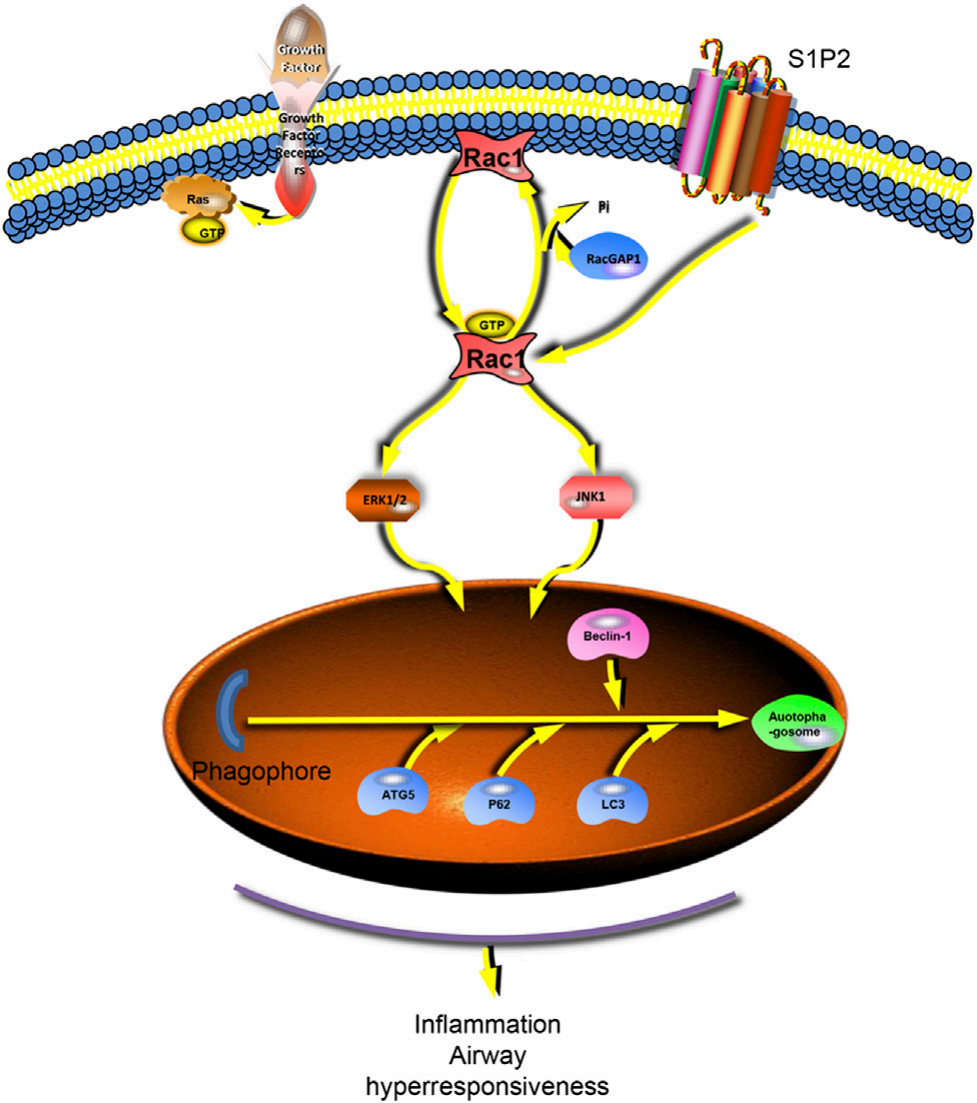
S1P2 receptor activates the downstream RAC1 and activates autophagy via Jnk and ERK1/2. The S1P2 receptor activates the downstream JNK and erk1/2 pathways by stimulating RAC1, further activating autophagy and generating inflammatory and airway remodeling responses in asthma.

In conclusion, our results showed that S1P was involved in the development of asthma, which had a significant role in both acute inflammation and chronic airway remodeling models of asthma. The action mechanism is related to the activation of S1PR2 receptors. The S1P2 receptor activation can further activate the RAC1/JNK pathway and may induce the onset of autophagy (Figure 7).

## Data Availability

The raw data supporting the conclusions of this article will be made available by the authors, without undue reservation.
